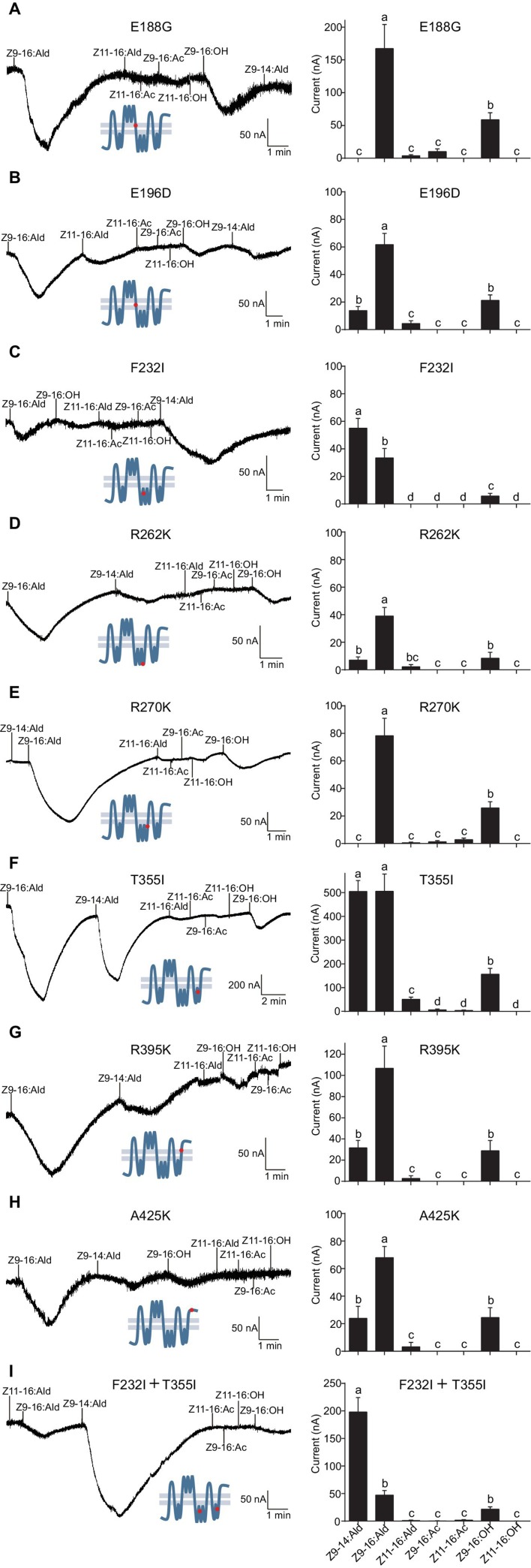# Correction: Two single-point mutations shift the ligand selectivity of a pheromone receptor between two closely related moth species

**DOI:** 10.7554/eLife.42513

**Published:** 2018-10-10

**Authors:** Ke Yang, Ling-Qiao Huang, Chao Ning, Chen-Zhu Wang

Yang K, Huang L-Q, Ning C, Wang C-Z. 2017. Two single-point mutations shift the ligand selectivity of a pheromone receptor between two closely related moth species. *eLife* **6**:e29100. doi: 10.7554/eLife.29100.

Published 24, October 2017

We discovered an error in predicting HassOr14b secondary structure through the TOPCONS webserver. This resulted in a wrong predicted secondary structure of HassOr14b in Figure 6, which was subsequently cited as the sketch maps of this structure in Figure 7 and Figure 8. All these figures have been updated to reflect this correction, and the expressions related to this error in the text have been corrected (The changes are underlined). The corrections have nothing to do with the experimental data and do not substantially change the conclusions.

In the Abstract:

1. “Two amino acids located in the *transmembrane* domains F232I and T355I together determine the functional difference between the two orthologs.”

Replaces the following text:

“Two amino acids located in the *intracellular* domains F232I and T355I together determine the functional difference between the two orthologs.”

In the Introduction:

1. “We used a series of regional replacements and single-point mutations, coupled with functional analyses, to demonstrate that two single-point mutations located in the *transmembrane* regions of the molecule together determine their ligand selectivity.”

Replaces the following text:

“We used a series of regional replacements and single-point mutations, coupled with functional analyses, to demonstrate that two single-point mutations located in the *intracellular* regions of the molecule together determine their ligand selectivity.”

In the Discussion:

1. “Its ortholog HarmOr14b is specific for Z9-14:Ald in *H. armigera* and we further demonstrate that two single-point mutations, F232I and T355I, located in the *transmembrane* domains of the receptor, together determine the functional shift between orthologs in the two closely related species.”

Replaces the following text:

“Its ortholog HarmOr14b is specific for Z9-14:Ald in *H. armigera* and we further demonstrate that two single-point mutations, F232I and T355I, located in the *intracellular* domains of the receptor, together determine the functional shift between orthologs in the two closely related species.”

2. “Two amino acids located in the transmembrane domains together determine the OR selectivity.”

Replaces the following text:

“Two amino acids located in the *intracellular* domains together determine the OR selectivity.”

3. “*Both of these amino acids reside within two of the TMDs, which agrees with that the associated TMDs and ECLs constitute the ligand-binding domain of ORs (Hughes et al., 2014; Leary et al., 2012).*”

Replaces the following text:

“*It is for the first time to find that the two mutation sites in the intracellular domains (ICDs) rather than in the TMDs and ECLs were involved in determination of ligand selectivity. We suggest two possible explanations for the role of ICDs. First, the binding site of ligand-specific ORs, such as PRs, may have a complex structure, which involves TMDs (Leary et al., 2012), ECLs (Hughes et al., 2014) and ICDs. Alternatively, ICDs may be involved in the specific interactions of the PR with the related G proteins. To relay the signal into the cell interior, binding of an extracellular molecule to an OR is tightly followed by binding of the receptor to a trimeric G protein inside the cell (Ignatious Raja et al., 2014; Wicher et al., 2008).*”

4. “We systematically identify two single-point mutations, F232I and T355I, located in the *transmembrane* regions of HassOr14b that together determine the functional shift to its ortholog, HarmOr14b, in *H. armigera*.”

Replaces the following text:

“We systematically identify two single-point mutations, F232I and T355I, located in the *intracellular* regions of HassOr14b that together determine the functional shift to its ortholog, HarmOr14b, in *H. armigera*.”

In the References:

The following two references have been removed as the related citations in the text were deleted:

Ignatious Raja JS, Katanayeva N, Katanaev VL, Galizia CG. 2014. Role of Go/i subgroup of G proteins in olfactory signaling of *Drosophila melanogaster. European Journal of Neuroscience* **39**: 1245-1255.

Wicher D, Schäfer R, Bauernfeind R, Stensmyr MC, Heller R, Heinemann SH, Hansson BS. 2008. *Drosophila* odorant receptors are both ligand-gated and cyclic-nucleotide-activated cation channels. *Nature* **452**: 1007-1012.

The corrected Figure 6 is shown here:

**Figure 1:**
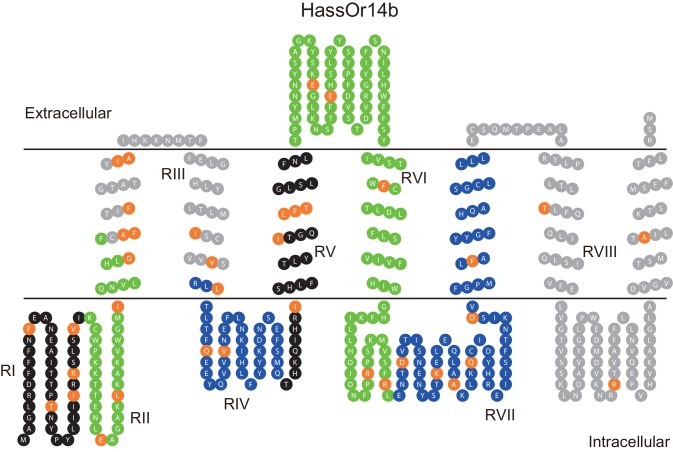


The originally published Figure 6 is also shown for reference:

**Figure 2:**
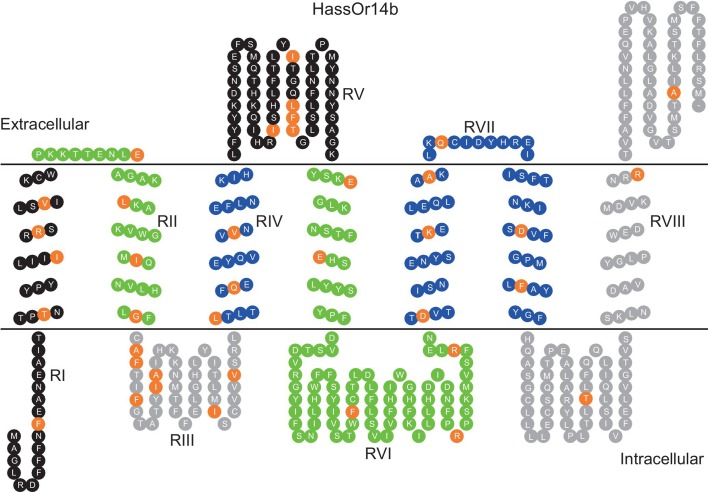


The updated Figure 7 is shown here:

**Figure 3:**
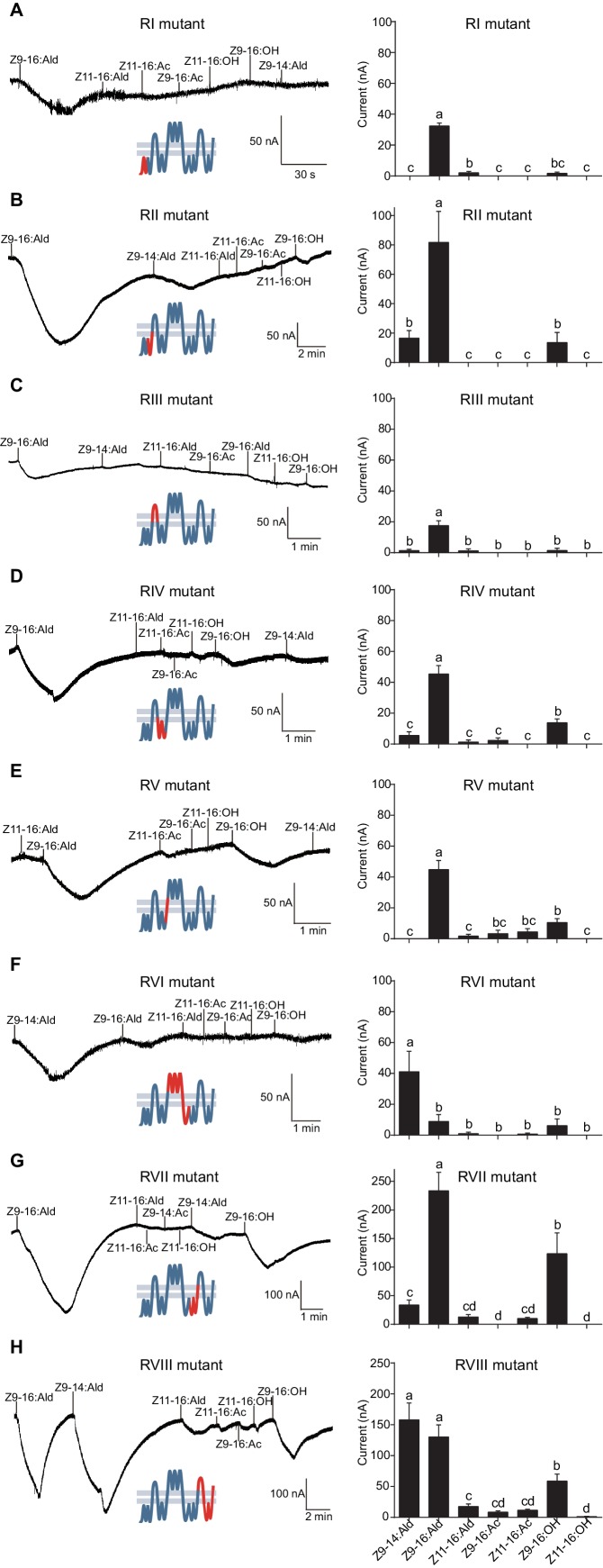


The originally published Figure 7 is also shown for reference:

**Figure 4:**
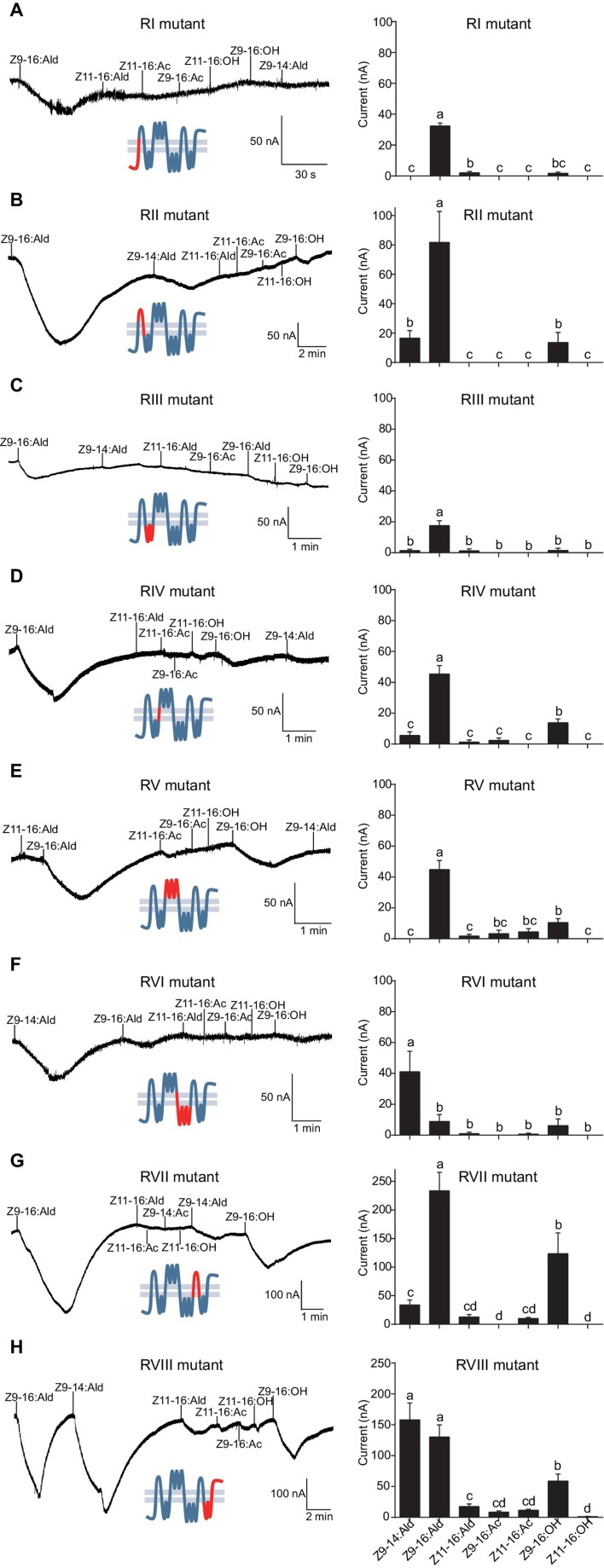


The updated Figure 8 is shown here:

**Figure 5:**
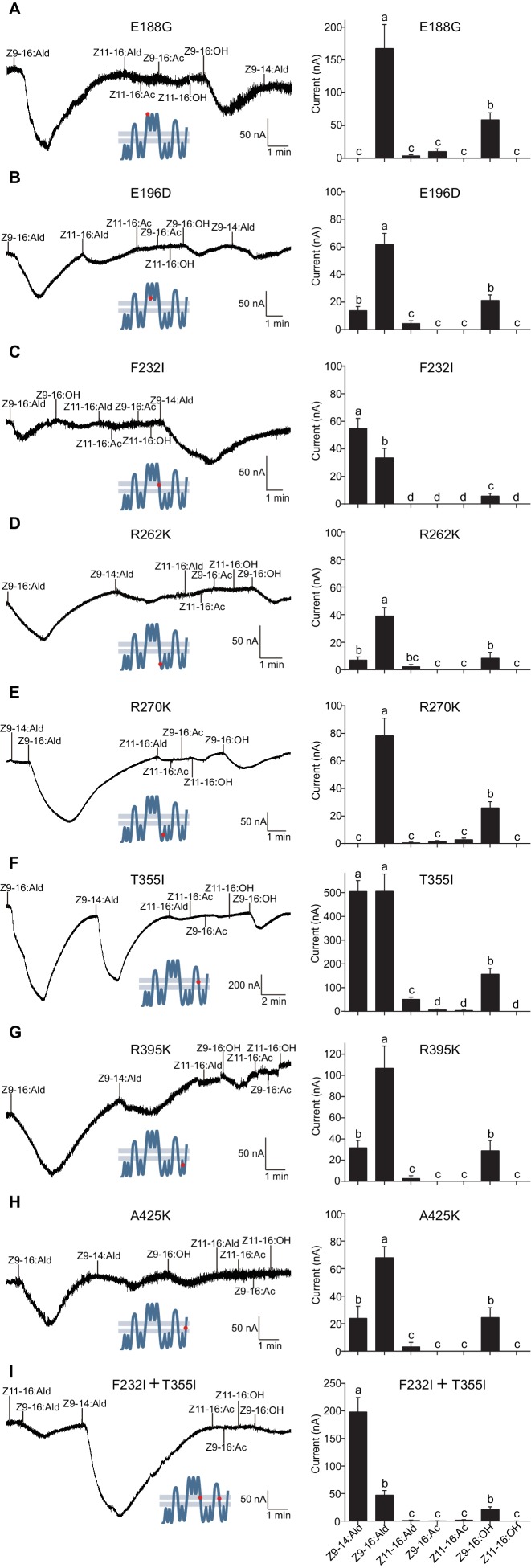


The originally published Figure 8 is also shown for reference:

**Figure 6:**