# Supplementary material for: Two single-point mutations shift the ligand selectivity of a pheromone receptor between two closely related moth species
Source: eLife. 2017 Oct 24;6:e29100. doi: 10.7554/eLife.29100 (PMC5673308; doi:10.7554/eLife.29100)
Supplement: Supplementary file 1. [file elife-29100-supp1.docx]

**Supplementary file 1.** Primers used for qPCR, *in situ* hybridization (*In situ*), *Xenopus* oocytes expression (Xe) and amino acid mutation (Mut).

| **Purposes** | **Primer Names** | **Sequences (5'–3')** |
| --- | --- | --- |
| qPCR | HassOr6-qF: | TGTCAGATGTGTTTGGGCGGA |
|  | HassOr6-qR: | GCTTGAGCCGTCATCTGCGA |
|  | HassOr11-qF: | ACGTGAAGGCTGGCGGATTG |
|  | HassOr11-qR: | ACGTGCGCAGAAATGCGAAG |
|  | HassOr13-qF: | TCCTGCAAACGCGACATACGA |
|  | HassOr13-qR: | TCGTGAGGTCCACAACGCAA |
|  | HassOr14-qF: | GTCGGAAGTGTTCGGGCCAA |
|  | HassOr14-qR: | AGCCTCCACAGTCACTTGCG |
|  | HassOr14b-qF: | CGGTTTTCACCAGGCAAGCG |
|  | HassOr14b-qR: | GTAAATAGCGGGCCAGGGCT |
|  | HassOr15-qF: | TGGCATCACAACACCCACAG |
|  | HassOr15-qR: | GGTCAACCAGATCTTTGCCCG |
|  | HassOr16-qF: | TCGCAGATGACAGCCCAAGC |
|  | HassOr16-qR: | GTCGCTCTCGCTTCCAACCA |
|  | HarmOr6-qF | CCGATGTGTTTGGGCCGATG |
|  | HarmOr6-qR | TTGGGCCGTCATCTGCGAAC |
|  | HarmOr11-qF | GCCGTGTTGCTGAGGGATTG |
|  | HarmOr11-qR | CTCGAGAAGCAGCAAGCACC |
|  | HarmOr13-qF | CTGGCCGAAGACTCCGAACG |
|  | HarmOr13-qR | CGGCTCCACCGACAAGAGTG |
|  | HarmOr14-qF | GGCTTACCGTGGGAAGACATGG |
|  | HarmOr14-qR | CCCAACGCCTTCACATGCAC |
|  | HarmOr14b-qF | TTCCACCAGGCAAGCGGATG |
|  | HarmOr14b-qR | AGTGGTAAATAGCGGGCCAGG |
|  | HarmOr15-qF | ACTATGGCTTTCACCAGGCGAG |
|  | HarmOr15-qR | ATAACGGACCAGAGCAGCCAC |
|  | HarmOr16-qF | AGACTCACCGTGCTGTCCAC |
|  | HarmOr16-qR | CCGGCGGCGTAGTTACTGT |
|  | Hass-Harm-18s-qF | CGTTGCTGGGAAGTTGACCA |
|  | Hass-Harm-18s-qR | CTTCCGCAGGTTCCCCTACG |
| *In situ* | HassOr14b-proF | GGCAGGGTTGCGTGATTTTT |
|  | HassOr14b-proR | TGCTCCAGCTCTTTGGTTGA |
|  | HassOr16-proF | CCCGAGTCGCCAAAGTTCT |
|  | HassOr16-proR | CTTGGGCTGTCATCTGCGAA |
|  | HassOr6-proF | TTAGGGGAACCCGAAAGTGC |
|  | HassOr6-proR | TACGAACAACATCCGCCCAA |
| Xe | HassOr6-xeF: | GAATTCgccaccATGAGCTTTAGAAAATTTCT |
|  | HassOr6-xeR: | CTCGAGTCACATACTGCGTAGAAAGGTGAAAT |
|  | HassOr14b-xeF: | GAATTCgccaccATGGCAGGGTTGCGTGATTT |
|  | HassOr14b-xeR: | TCTAGATTACATACTGCGTAGGAAGGTGA |
|  | HassOr16-xeF: | GAATTCgccaccATGGGTCTTCGCCAATTTC |
|  | HassOr16-xeR: | TCTAGATTACATACTCCTTAAAAACAT |
|  | HassOrco-xeF: | GAATTCgccaccATGATGACCAAAGTGAAGGCCC |
|  | HassOrco-xeR: | TCTAGATTACTTGAGTTGTACCAACACCAT |
|  | HarmOr14b-xeF: | CTCGAGgccaccATGGCAGGGTTGCGTGATTT |

| **Purposes** | **Primer Names** | **Sequences (5'–3')** |
| --- | --- | --- |
| Xe | HarmOr14b-xeR: | TCTAGATTACATACTGCGTAGGAAGG |
|  | HarmOrco-xeF: | GAATTCgccaccATGATGACCAAGGTGAAGGCC |
|  | HarmOrco-xeR: | TCTAGATTACTTGAGTTGTACCAACAC |
| Mut | HarmOr14b-RI-1-R: | GCCTCGAGGTTTTCCGTAGTTTTTTTAGGCCAAC |
|  | HassOr14b-RI-2-F: | CACTTATCAAATGTTGGCCTAAAAAAACTACGG |
|  | HassOr14b-RII-1-R: | GCCGCGAGATTTTCCGTAGTTTTTTTAGGCCAAC |
|  | HarmOr14b-RII-2-F: | CAGTTATCAAATGTTGGCCTAAAAAAACTACGG |
|  | HarmOr14b-RII-2-R: | CGTGAACGCACAGAATGCCAAATGTAAAAC |
|  | HassOr14b-RII-3-F: | CAAAATGTTTTACATTTGGCATTCTGTGCGTTC |
|  | HassOr14b-RIII-1-R: | CAATCGTTAACACACAGAATCCCAAATGTAAAAC |
|  | HarmOr14b-RIII-2-F: | TACAAAATGTTTTACATTTGGGATTCTGTGTG |
|  | HarmOr14b-RIII-2-R: | GTAAGCGTTAGTAATCGGGAAAAGACAACGCAAC |
|  | HassOr14b-RIII-3-F: | GTCTTTTCCCGATTACTAACGCTTACTTTTC |
|  | HassOr14b-RIV-1-R: | AGTAGCTAATCGGGAAACGACAACGCAACTTATC |
|  | HarmOr14b-RIV-2-F: | GTTGCGTTGTCGTTTCCCGATTAGCTACTCTTAC |
|  | HarmOr14b-RIV-2-R: | CCGATGAATTTGCTTGTGGGTC |
|  | HassOr14b-RIV-3-F: | CAATAAAATCCATCTCTTCTAC |
|  | HassOr14b-RV-1-R: | TGAATTGTCTTTGTAGTAGAAGAGATG |
|  | HarmOr14b-RV-2-F: | AATGAGTTTCTTAATAAAATCCATCTCTTCTAC |
|  | HarmOr14b-RV-2-R: | CCAGCAGAATAATTGTTGTACATAGGAGTTAAATTG |
|  | HassOr14b-RV-3-F: | TGGTCTCTCACTCTTCAATTTAACTCCTATGTA |
|  | HassOr14b-RVI-1-R: | CCAGCAGAATAATTGTTATACATAGGAGTTAG |
|  | HarmOr14b-RVI-2-F: | GGTCTCTCACTCTTCAATCTAACTCCTATG |
|  | HarmOr14b-RVI-2-R: | CGTGACATCAGTGTTTTCTAACTTAAAGCTAACC |
|  | HassOr14b-RVI-3-F: | GCTTTAAGTTAGAAAACACTGATGTCACG |
|  | HassOr14b-RVII-1-R: | AGTGACGTTAGTATTTTCTAACCTGAAGCTAACC |
|  | HarmOr14b-RVII-2-F: | GCTTCAGGTTAGAAAATACTAACGTCACTATTTC |
|  | HarmOr14b-RVII-2-R: | GTAAGTGGTAAATAGCGGGCCAG |
|  | HassOr14b-RVII-3-F: | CTACTCCTGGAGTGTTCAC |
|  | HassOr14b-RVIII-1-R: | GTAAGTGGTAAATAGCGGGCCAG |
|  | HarmOr14b-RVIII-2-F: | CTACTCCTGGAGTGTTCACAAATG |
|  | E188G-1-R: | GTGTTCGAACGTTGAGTTTTTCAATCCCCCTTTG |
|  | E188G-2-F: | ATTATTCTGCTGGAAAATACAGCAAAGGGGGATTG |
|  | E196D-1-R: | GGATAGGAGTAGTATAGAGAGTGATCGAACGTTG |
|  | E196D-2-F: | AGAGGGATTGAAAAACTCAACGTTCGATCACTCTC |
|  | F232I-1-R: | CCGATAAAAAGAGATCGAGGGTACAGATCCAAG |
|  | F232I-2-F: | CATACATAGTATCAACTTGGATCTGTACCCTCGATC |
|  | R262K-1-R: | CTAACCTGAAGCTAACCATTTTTGATGGCTTTGG |
|  | R262K-2-F: | CTACTTCATGATATCGACAATTTTCCAAAGCCATC |
|  | R270K-1-R: | AATCGTGACATCAGTGTTTTCTAACTTGAAGC |
|  | R270K-2-F: | CAAGGCCATCAAAAATGGTTAGCTTCAAGTTAG |
|  | T355I-1-R: | CGACAGTTGGATGAGTTGCTGAAATAAGATTAATG |
|  | T355I-2-F: | GGCCCGCTATTTACCACTTACATTAATCTTATTTC |
|  | R395K-1-R: | CTGTACATTCAATAGGAAGAAGGCCACAGTCTTCC |
|  | R395K-2-F: | GGGAGGACATGGATGTAAAAAATAGGAAGACTGTG |
|  | A425K-1-R: | GCACAGGTTCCTGTACATTCAATAGGAAGAAGGCCAC |
|  | A425K-2-F: | GACTGTGGCCTTCTTCCTATTGAATG |

F: forward primer; R: reverse primer. The underlined indicate restriction recognition sites, the minuscule indicate Kozak sequence.
