# Supplementary material for: Two single-point mutations shift the ligand selectivity of a pheromone receptor between two closely related moth species
Source: eLife. 2017 Oct 24;6:e29100. doi: 10.7554/eLife.29100 (PMC5673308; doi:10.7554/eLife.29100)
Supplement: Supplementary file 2. [file elife-29100-supp2.docx]

**Supplementary file 2.** The accession numbers of all PRs and Orcos used in phylogenetic analysis.

| **Categories** | **Species** | | **Gene Names** | | **Accession Numbers** | | |
| --- | --- | --- | --- | --- | --- | --- | --- |
| PRs | *A. segetum* | | *AsegOr1* | | AGS41441 | | |
|  |  | | *AsegOr3* | | AGS41442 | | |
|  |  | | *AsegOr4* | | AGS41443 | | |
|  |  | | *AsegOr5* | | AGS41444 | | |
|  |  | | *AsegOr6* | | AGS41445 | | |
|  |  | | *AsegOr7* | | AGS41446 | | |
|  |  | | *AsegOr8* | | AGS41447 | | |
|  |  | | *AsegOr9* | | AGS41448 | | |
|  |  | | *AsegOr10* | | AGS41449 | | |
|  | *H. armigera* | | *HarmOr6* | | JX982525 | | |
|  |  | | *HarmOr11* | | ACF32965 | | |
|  |  | | *HarmOr13* | | ACJ12370 | | |
|  |  | | *HarmOr14* | | JX982530 | | |
|  |  | | *HarmOr14b* | | JX982531 | | |
|  |  | | *HarmOr15* | | AIG51863 | | |
|  |  | | *HarmOr16* | | ACS45306 | | |
|  | *H. assulta* | | *HassOr6* | | JX982539 | | |
|  |  | | *HassOr11* | | ACS45308 | | |
|  |  | | *HassOr13* | | ACS45307 | | |
|  |  | | *HassOr14* | | AHI44516 | | |
|  |  | | *HassOr14b* | | JX982544 | | |
|  |  | | *HassOr15* | | AJD81553 | | |
|  |  | | *HassOr16* | | ACS45309 | | |
|  | *H. virescens* | | *HvirOr6* | | CAD31948 | | |
|  |  | | *HvirOr11* | | CAG38112 | | |
|  |  | | *HvirOr13* | | CAG38114 | | |
|  |  | | *HvirOr14* | | CAG38115 | | |
|  |  | | *HvirOr15* | | CAG38116 | | |
|  |  | | *HvirOr16* | | CAG38117 | | |
|  | *M. separata* | | *MsepOr1* | | BAG71414 | | |
|  |  | | *MsepOr3* | | BAG71423 | | |
|  | *S. inferens* | | *SinfOr21* | | KC960468 | | |
|  |  | | *SinfOr27* | | KC960474 | | |
|  |  | | *SinfOr29* | | KC960476 | | |
|  | *S. littoralis* | | *SlittOr6* | | ACL81183 | | |
|  |  | | *SlittOr13* | | ACL81181 | | |
|  |  | | *SlittOr11* | | ACL81180 | | |
|  |  | | *SlittOr16* | | ACL81182 | | |
| **Categories** | | **Species** | | **Gene Names** | | **Accession Numbers** |  |
| PRs | | *S. litura* | | *SlituOr6* | | AGI96748 |  |
|  | |  | | *SlituOr11* | | AGI96749 |  |
|  | |  | | *SlituOr13* | | AGI96750 |  |
|  | |  | | *SlituOr16* | | AGI96751 |  |
|  | | *S. exigua* | | *SexiOr6* | | AGH58119 |  |
|  | |  | | *SexiOr11* | | AGH58120 |  |
|  | |  | | *SexiOr13* | | AGH58121 |  |
|  | |  | | *SexiOr16* | | AGH58122 |  |
| Orcos | | *A. segetum* | | *AsegOrco* | | KC526964 |  |
|  | | *H. virescens* | | *HvirOrco* | | CAD31851 |  |
|  | | *H. armigera* | | *HarmOrco* | | HQ186284 |  |
|  | | *H. assulta* | | *HassOrco* | | HQ186285 |  |
|  | | *M. separata* | | *MsepOrco* | | BAG71415 |  |
|  | | *S. littoralis* | | *SlittOrco* | | ABQ82137 |  |
|  | | *S. inferens* | | *SinfOrco* | | KC960454 |  |
|  | | *S. exigua* | | *SexiOrco* | | AAW52583 |  |
|  | | *S. litura* | | *SlituOrco* | | ABH10019 |  |
